# Remarks on Nosology

**Published:** 1810-02

**Authors:** 


We have been so entertained with the pleasantry of the following short letter, that we insert the whole as received, even with the droll postscript.

### *To the Editors of the Medical and Physical Journal.*

GENTLEMEN,

YOUR answer to the letter signed an Old Correspondent, (sent you in October) in your Journal of November, seems to have arisen from a complete misunderstanding of the meaning, purport, and intent thereof. There were no anonymous observations in it upon any gentleman whatever. The inquiry made, both then and in the former letter addressed to you, was simply this, (and which, from your extensive acquaintance with authors, was thought not difficult to be answered by you) where can I find the history of, diagnosis, prognosis, and methodus medendi of a disease I see every month, or nearly so, called Gastrodynia? If any author has treated of it, the gentleman who uses the term, should be respectfully requested to give him to the world; or the term, together with some others, should be given up.

Ecce iterum Martinus Scriblerus.

On the cover of your Journal, this day received, as yet not cut open, I see advertised, a second edition of the New London Pharmacopœia, *revised* and *corrected*. Surely the first cannot *as yet* require *revision* and *correction!* Alas, what am I to do? I have bought the first, and cannot afford to buy the second already; is there no little sheet or list printed of these Errata and Corrigenda, to bind up with the first edition? that's somewhat hard upon us poor fellows, whose guineas and half guineas do not roll in upon us so fast as to require a leather lining to the waistcoat pocket, (a thing I have heard which was formerly much used); no, we must, I perceive, remain in ignorance,

 because because we are poor; possibly this new edition may contain a direction how to make the old Sal. Amarus (sulphate of magnesia, I believe it is now to be called) for I cannot find it in the first, nor can I discover it in the list of articles omitted.

In your Intelligence for this month, you mention that common spirit of turpentine has been recently administered, with good effect, in cases of tape worm. I have prescribed it for many years, exactly as there recommended, and have brought away, or caused to be expelled, many feet of that worm at a time. I always took care to ascertain tolerable clearly, and indeed, sometimes with certainty, what the case was before I ordered it. In some cases it has been necessary to guard the kidneys from its effects, by ordering plenty of emollient drinks to be given at the same time. I regret I cannot say where I got the remedy, it was many years ago. I cannot help thinking it was from Dr. Donald Monro's Medical and Pharmaceutical Chemistry.

In a letter I received last week from Edinburgh, mention is made, that Mr. Walker, author of the Archives of Universal Science, has produced a paper, in which he describes a part in the brain hitherto unnoticed and unknown; I hope your next month's Journal will give us some account of it.

I am, &c.

An Old Correspondent.

P. S. Pray do not forget me next month; don't quibble nor mistake my plain meaning, in answer to my former letters, and first part of this.

